# Progesterone-induced endometrial thickness changes do not affect singleton neonatal outcomes in frozen embryo transfer: a retrospective analysis of 6,331 newborns

**DOI:** 10.1186/s12884-026-09391-9

**Published:** 2026-06-20

**Authors:** Jing Ye, Jie Zhang, Tong Du, Sha Yu, Yanwen Zhu, Hongyuan Gao, Yali Liu, Qiujiu Chen, Yanping Kuang

**Affiliations:** Department of Assisted Reproduction, Shanghai Ninth People’s Hospital, Shanghai Jiaotong University School of Medicine, 639 Zhizaoju Rd, Shanghai, 200001 China

**Keywords:** Birthweight, frozen-thawed embryo transfer, endometrial thickness change, Neonatal outcomes

## Abstract

**Background:**

To evaluate whether progesterone-induced changes in endometrial thickness (EMT) affect singleton infant outcomes during frozen-thawed embryo transfer (FET) cycles.

**Methods:**

This retrospective study analyzed 6,331 singleton live births following frozen-thawed Day 3 embryo transfers. EMT was measured via transvaginal ultrasound one day before progesterone initiation and on the day of FET. Participants were grouped by EMT change: increase, decrease, or stable. Primary outcomes included mean birthweight, low birthweight (LBW), and small-for-gestational-age (SGA) status. Associations between EMT changes and neonatal outcomes were evaluated using multivariable linear and logistic regression.

**Results:**

No significant differences in mean birthweight were observed among the three groups (3,355.30 ± 502.69 g vs. 3,351.30 ± 474.79 g vs. 3,344.26 ± 514.54 g; *p* = 0.753). Compared with the EMT stable group, the EMT decreased group showed no significant increase in LBW (1.1%; aOR 1.645, 95% CI 0.818–3.307) or SGA (2.7%; aOR 1.141, 95% CI 0.783–1.662). Similarly, the EMT increased group showed no significant association with LBW (aOR 1.310, 95% CI 0.723–2.375) or SGA (aOR 0.912, 95% CI 0.660–1.261). Multiple linear regression confirmed that gestational age and infant gender significantly influenced birthweight, while EMT change did not.

**Conclusions:**

EMT may increase, decrease, or remain stable after progesterone administration in FET cycles. However, these changes do not demonstrate an independent association with adverse perinatal outcomes in term singleton infants.

**Supplementary Information:**

The online version contains supplementary material available at https://doi.org/10.1186/s12884-026-09391-9.

## Introduction

Since the advent of in vitro fertilization (IVF) in 1978, the use of assisted reproductive technologies (ART) has grown significantly, leading to steadily rising live birth rates worldwide. The potential health effects on ART-conceived children are now a major public concern. In general, most IVF offspring enjoy good overall health. However, IVF pregnancies are associated with an increased risk of adverse perinatal outcomes, such as preterm birth, low birth weight, and small for gestational age [1, 2]. The causes of poor outcomes in ART singletons are multifactorial. Research by Pinborg A et al. identified subfertility as a key risk factor for adverse perinatal outcomes in ART-conceived singletons. Their study also found that, even among siblings from the same mother, ART singletons had worse outcomes than those conceived naturally. The authors suggested that ovarian stimulation and IVF laboratory procedures—including embryo culture and cryopreservation—may further contribute to these results [2]. Emerging evidence also indicates that various IVF-related factors, such as patient characteristics, ovarian stimulation protocols, insemination techniques, cryopreservation methods, and embryo culture medium, may play a role in these suboptimal outcomes [3–8].

Assessment of birthweight is standard in ART populations, as it critically indicates neonatal health due to strong links with first-year mortality, childhood developmental issues, and increased adult disease risk [9]. Moreover, low birth weight relative to gestational age is associated with higher risks of coronary heart disease, stroke, hypertension, and type 2 diabetes [10].

The measurement of endometrial thickness by transvaginal ultrasound is widely considered a prognostic marker of endometrial receptivity, though its clinical significance remains debated [11–18]. In prior studies, it has generally been assessed either during ovarian stimulation in fresh embryo transfer cycles or during the proliferative phase before progesterone administration in frozen-thawed cycles. Strong evidence consistently shows that a decrease in peak endometrial thickness is significantly associated with lower pregnancy rates in both fresh and frozen-thawed embryo transfer cycles [11, 19–22]. Barker et al. assessed the link between luteal-phase endometrial thickness-measured on the day of embryo transfer—and pregnancy outcomes in an oocyte donation model [18]. The distinct physiology of the endometrium during the follicular and luteal phases, both in natural cycles and IVF, is well documented [23–25]. The endometrial pattern progresses sequentially from pattern A to B to C. While many studies have explored the relationship between these patterns and ART outcomes, the results remain inconsistent [25–29].

Most studies have focused on endometrial thickness at a single, static time point-often during the follicular/proliferative phase (e.g., on hCG trigger day or before progesterone administration) or in fresh embryo transfer cycles-rather than on its dynamic changes in response to progesterone, particularly in frozen embryo transfer cycles [30]. Luteal phase endometrial thickness remains an understudied predictor of neonatal outcomes. While Barker et al. examined luteal phase thickness in oocyte donation cycles, their analysis emphasized pregnancy establishment over long-term neonatal outcomes such as birthweight, low birthweight, or small-for-gestational-age status [30]. The link between progesterone-induced endometrial changes and neonatal health outcomes remains poorly understood. Although our institution has identified reduced endometrial thickness as an independent risk factor for preterm birth, low birth weight, and small-for-gestational-age infants in women with polycystic ovary syndrome [31], the impact of the degree or pattern of endometrial thickness change after progesterone administration on birthweight and other neonatal outcomes in singleton frozen-thawed embryo transfer cycles is still unclear. Our prior research in such cycles focused mainly on pregnancy rates and did not assess neonatal outcomes [32].

To date, little research has examined the link between progesterone-induced endometrial thickness (EMT) changes and neonatal outcomes. While our group previously found no impact of EMT alterations on pregnancy or live birth rates in frozen embryo transfer cycles, this study is the first large-scale analysis of progesterone-induced EMT changes and perinatal outcomes in singleton live births. Unlike earlier studies that measured EMT at a single proliferative-phase time point, we assessed dynamic EMT responses to progesterone-a distinct parameter reflecting secretory transformation rather than estrogen-driven proliferation.

## Methods

### Study design and patients

This retrospective cohort study was conducted at the Department of Assisted Reproduction, Ninth People’s Hospital, Shanghai Jiao Tong University School of Medicine. It included 6,331 women who underwent frozen embryo transfer (FET) cycles between January 2010 and December 2019. Inclusion criteria were age ≤ 40 years, body mass index < 30 kg/m², and the transfer of a day-3 embryo during an FET cycle. Exclusion criteria included uterine malformation, endometriosis, adenomyosis, and the presence of endometrial polyps or submucosal myomas detected by hysteroscopy that could affect implantation. The study protocol was approved by the hospital’s Ethics Committee, and the research adhered to the principles of the Declaration of Helsinki. As an observational study, no interventions were performed.

### Endometrial preparation protocol heterogeneity

This study included both natural cycle (NC) and estrogen-progesterone (EP) frozen embryo transfer (FET) cycles, which differ in their hormonal patterns. In NC cycles, endometrial development is driven by endogenous estradiol from a dominant follicle, with progesterone supplementation starting after ovulation. Conversely, EP cycles use exogenous oral estradiol (8 mg/day) to promote endometrial growth, followed by progesterone once endometrial thickness reaches ≥ 7 mm. The protocols vary in three key ways: (1) the type of estradiol exposure before progesterone (endogenous and fluctuating vs. exogenous and steady); (2) the presence of a corpus luteum (present in NC, absent in EP); and (3) the extent of luteal phase support (minimal in NC vs. intensive in EP).

### Treatment protocol

In the natural cycle frozen embryo transfer protocol, embryo transfer timing was determined by follicular size, endometrial thickness, and serum hormone levels. Transvaginal ultrasound monitoring commenced on cycle day 10. A single clinician performed all ultrasounds, measuring endometrial thickness and the mean diameter of the dominant follicle. Once the leading follicle reached approximately 18 mm and the endometrial thickness was at least 7 mm, serum progesterone, estradiol, and luteinizing hormone levels were assessed. If estradiol exceeded 150 pg/mL and progesterone was below 1 ng/mL, the subsequent steps depended on the LH level. For LH levels under 20 mIU/mL, 5,000 IU of hCG was administered that evening. Progesterone supplementation began three days after hCG, with a cleavage-stage embryo transferred two days later (five days post-hCG). If the serum LH concentration was ≥ 20 mIU/mL, hCG was given the same afternoon. Progesterone supplementation then started two days later, and embryo transfer was performed four days after hCG administration. Endometrial thickness on the day of hCG injection was recorded via transvaginal ultrasound.

For the estrogen-progesterone regimen, oral micronized estradiol (8 mg daily) was started on cycle day 3. Endometrial thickness was measured by transvaginal ultrasound 12–14 days later. Once endometrial thickness reached ≥ 7 mm, progesterone supplementation began. Patients then started Fematon tablets (yellow; Abbott Healthcare Products B.V.)-two tablets twice daily, each containing 2 mg micronized estradiol and 10 mg dydrogesterone-alongside intravaginal progesterone soft capsules (200 mg twice daily; Laboratoires Besins International). Endometrial thickness on the day before starting progesterone was recorded via transvaginal ultrasound.

Detailed protocols for endometrial preparation in frozen embryo transfer (FET) cycles and methods for assessing endometrial thickness (EMT) are well-documented in the literature [21, 32, 33]. Frozen-thawed embryo transfer was conducted on the third day of progesterone support, with a maximum of two embryos transferred per cycle.

Due to differing hormonal environments between natural cycles (NC) and estrogen-progesterone (EP) cycles, we performed subgroup analyses by preparation protocol. NC cycles rely on endogenous hormones and a corpus luteum, whereas EP cycles use exogenous estrogen and progesterone without a corpus luteum [34]. Prior studies indicate higher birthweight in artificial versus natural cycles [35], suggesting potential effect modification by cycle type. We tested for interaction by including product terms (EMT change × cycle type) in multivariable models.

### EMT assessment

All ultrasound measurements were performed by an experienced senior technician using a GE Voluson E8 system (GE Healthcare, Austria). Transvaginal examinations employed standardized high-resolution equipment with 6 MHz endovaginal transducers. Imaging parameters were uniformly applied: the transducer frequency was fixed at 6 MHz for optimal uterine visualization; spatial resolution was maximized using harmonic imaging; gain was calibrated to clearly define the endometrial-myometrial interface without signal saturation; the field of view encompassed the full uterine axis from fundus to cervix; and image magnification was adjusted so the endometrium occupied ≥ 75% of the image height to ensure accurate measurement.

To ensure accuracy and reliability, endometrial thickness was assessed by expert physicians, each with at least seven years of experience in transvaginal ultrasonography (TVU). Measurements were taken using a 6-MHz vaginal transducer (Voluson; GE Healthcare, Austria). A standardized protocol at our center assigned each patient to one physician throughout the in vitro fertilization (IVF) cycle, ensuring consistent monitoring and minimizing discomfort from multiple operators. Endometrial thickness (EMT) was defined as the maximum distance between the endometrial and myometrial reflective interfaces in the midsagittal uterine plane.

Endometrial thickness (EMT) was measured as previously described [21, 32, 33]. It was assessed during the last transvaginal ultrasound before progesterone initiation—specifically, on hCG administration day in natural cycles or before progesterone start in estrogen-primed cycles. Another transvaginal ultrasound was performed on the morning of embryo transfer for all patients to confirm the absence of endometrial cavity fluid or other adverse conditions.

The absolute change in endometrial thickness (ΔEMT) was calculated as follows: Δ*EMT*=*EMT*_*ET day*_−*EMT*_*P*−1_, where *EMT*_*P*−1_ denotes the endometrial thickness (mm) on the day preceding progesterone initiation, and *EMT*_*ET day*_ represents the endometrial thickness (mm) on the day of embryo transfer. Based on ΔEMT, endometrial response was categorized as: EMT Decrease (Compaction) (ΔEMT ≤ − 1.0 mm), EMT stable (− 1.0 mm < ΔEMT < + 1.0 mm), or EMT Increase (Expansion) (ΔEMT ≥ + 1.0 mm).

### Outcome measures and definitions

Live birth was defined as delivery of a viable neonate at ≥ 24 weeks of gestation. In FET cycles, gestational age (GA) was calculated from the embryo transfer date, set as Day 17 for embryos cryopreserved on Day 3 [36]. Preterm and very preterm births referred to delivery before 37 and 32 completed weeks, respectively. Small for gestational age (SGA) and very small for gestational age (VSGA) were defined as birthweights below the 10th and 3rd percentiles for GA, respectively. Large for gestational age (LGA) indicated a birthweight above the 90th percentile, and very large for gestational age (VLGA) above the 97th percentile. Birthweight categories were: low birthweight (LBW) as < 2500 g, very low birthweight (VLBW) as < 1500 g, high birthweight (HBW) as > 4000 g, and very high birthweight (VHBW) as > 4500 g. Birthweight was adjusted for GA and neonatal gender using a Z-score based on a published formula [21], with percentiles and Z-scores referenced to a Chinese singleton newborn population stratified by gender and GA [37]. Pregnancy and neonatal outcomes data were obtained from electronic medical records.

Gestational age at delivery was prespecified as a potential effect modifier for three reasons: (1) preterm birth involves distinct mechanisms (e.g., infection or placental insufficiency) that may mask subtle endometrial effects; (2) birthweight is closely tied to gestational age, and combining term and preterm infants could hide EMT-related growth restriction; and (3) the causes of SGA differ in term (often placental/fetal) versus preterm (frequently iatrogenic) pregnancies. Analyses were stratified at 37 weeks.

### Statistical analysis

Baseline characteristics and neonatal outcomes were compared using one-way ANOVA for continuous variables and χ² or Fisher’s exact tests for categorical variables, as appropriate. Multivariable logistic regression was used to evaluate associations between changes in EMT and neonatal outcomes, while multiple linear regression assessed the independent effect of EMT change on birthweight. Before model fitting, variance inflation factors (VIF) were calculated for all covariates to check multicollinearity, with a VIF < 5 considered acceptable (Supplement Table 1). All multivariable models adjusted for maternal age, BMI, paternal age, parity, infertility duration, history of prior SGA delivery, number of embryos transferred, endometrial preparation type, frozen embryo transfer cycle rank, pregnancy complications (including GDM, hypertension, pre-eclampsia, or placenta previa), newborn sex, and gestational age at birth. All covariates were retained in the final models.

The completeness of the dataset was assessed prior to analysis. Missing data were minimal (< 1% for all covariates) and occurred sporadically for paternal age (*n* = 12, 0.19%) and infertility duration (*n* = 8, 0.13%). Given the low proportion of missingness, complete case analysis was employed. Sensitivity analyses using multiple imputation by chained equations (MICE) with 20 imputed datasets confirmed that the primary results were robust to missing data assumptions (data not shown).

The stable EMT group served as the reference for comparison. Statistical significance was set at *p* < 0.05. All analyses were performed using SPSS (version 25.0).

## Results

### Baseline characteristics

The final analytical cohort included 6,331 singleton live births meeting the inclusion criteria with complete follow-up. Based on endometrial thickness trajectory, the cohort was divided into three groups: decrease (*n* = 1,746), stable (*n* = 1,750), and increase (*n* = 2,835) (Table 1). Maternal age, body mass index, paternal age, cause of infertility, history of prior small-for-gestational-age birth, and frozen-thawed embryo transfer cycle order showed no significant differences among groups. Significant differences were observed, however, in parity, duration of infertility, endometrial preparation protocol, and number of embryos transferred (Table 1).

**Table 1:** Baseline demographic and cycle characteristics according to progesterone-induced endometrial thickness change

	EMT Decrease (*n* = 1746)	EMT stable (*n* = 1750)	EMT Increase (*n* = 2835)	*P* value
Maternal age (y)	31.82 ± 3.87	31.66 ± 3.76	31.66 ± 3.74	0.329^a^
Maternal BMI (kg/m^2^)	21.58 ± 2.94	21.53 ± 2.85	21.54 ± 3.01	0.854^a^
Paternal age (y)	33.92 ± 5.03	33.79 ± 4.85	33.71 ± 4.93	0.361^a^
Infertility cause				0.142^b^
Female	977(56.0)	1006(57.5)	1540(54.3)	
Male	252(14.4)	263(15.0)	428(15.1)	
Mixed	367(21.0)	316(18.1)	608(21.4)	
Unexplained	150(8.6)	165(9.4)	259(9.1)	
Parity, n (%)				0.000^b^
First	874(50.1)	941(53.8)	1593(56.2)	
High order	872(49.9)	809(46.2)	1242(43.8)	
Infertility duration (y)	3.64 ± 2.67	4.03 ± 2.78	3.65 ± 2.65	0.000^a^
FET cycle rank, n (%)				0.185^b^
First	1097(62.8)	1130(64.6)	1857(65.5)	
High order	649(37.2)	620(35.4)	978(34.5)	
FET endometrial preparation, n (%)				0.000^b^
Ovulatory cycle	1110(63.6)	1156(66.1)	1962(69.2)	
Artificial cycle	636(36.4)	594(33.9)	873(30.8)	
*EMT*_*P*−1_ (mm)	11.4 ± 2.2	11.4 ± 2.5	11.2 ± 1.8	0.345^a^
*EMT*_*ET day*_ (mm)	10.0 ± 2.0	11.4 ± 2.5	12.4 ± 2.3	< 0.001^a^
Number of embryos transferred, n (%)				0.029^b^
1	150(8.6)	164(9.4)	205(7.2)	
2	1596(91.4)	1586(90.6)	2630(92.8)	
Previous SGA newborns, n (%)	8(0.5)	3(0.2)	11(0.4)	0.314^c^
Newborn gender, n (%)				0.599^b^
Female	852(48.8)	866(49.5)	1360(48.0)	
Male	894(51.2)	884(50.5)	1475(52.0)	
Gestational age (weeks), n (%)				0.367^b^
< 37	129(7.4)	110(6.3)	184(6.5)	
≥ 37	1617(92.6)	1640(93.7)	2651(93.5)	
Pregnancy-related complications	156(8.9)	173(9.9)	238(8.4)	0.229^b^
Birth weight (g)	3355.30 ± 502.69	3351.30 ± 474.79	3344.26 ± 514.54	0.753^a^
Z-score	0.43 ± 1.04	0.38 ± 1.04	0.34 ± 1.07	0.023^a^

Data are presented as mean ± SD or n (%)

*SGA* small-for-gestational-age, *EMT*_*P*−1_ denotes the endometrial thickness (mm) on the day preceding progesterone initiation, *EMT*_*ET day*_ represents the endometrial thickness (mm) on the day of embryo transfer

^a^One-way ANOVA; ^b^Pearson chi-square test; ^c^Fisher’s exact test

### Main outcome

Regarding the primary outcome of birth weight, no significant difference in mean birth weight was observed across the three groups (3,355 ± 503 g vs. 3,351 ± 475 g vs. 3,344 ± 515 g; *p* = 0.753) (Table 2). However, Z-scores showed significant between-group variation, with the increase group (0.34 ± 1.07) having lower values than both the decrease (0.43 ± 1.04) and stable groups (0.38 ± 1.04; *p* = 0.023) (Table 1). Among term infants (≥ 37 weeks), EMT change was not significantly associated with low birth weight (LBW) or small for gestational age (SGA), as all adjusted odds ratio (aOR) 95% confidence intervals included 1.0 (Table 3). In preterm infants (< 37 weeks), birth weight differed significantly among the groups (*p* = 0.025), but the sample size was limited (*n* = 423), and no significant associations were found between EMT change and LBW or SGA (Table 4).

**Table 2:** Neonatal birthweight and weight-for-gestational-age categories by endometrial thickness change group

	EMT Decrease (*n* = 1746)	EMT stable (*n* = 1750)	EMT Increase (*n* = 2835)	*P* value
Newborn gender, n (%)				0.599^b^
Female	852(48.8)	866(49.5)	1360(48.0)	
Male	894(51.2)	884(50.5)	1475(52.0)	
Mean birthweight (g)	3355.30 ± 502.69	3351.30 ± 474.79	3344.26 ± 514.54	0.753^a^
Normal weight, n (%)	1513(86.7)	1550(88.6)	2476(87.3)	0.248^b^
LBW, n (%)	64(3.7)	63(3.6)	105(3.7)	
HBW, n (%)	145(8.3)	110(6.3)	201(7.1)	
VSGA, n (%)	26(1.5)	33(1.9)	49(1.7)	0.133^b^
SGA, n (%)	47(2.7)	55(3.1)	111(3.9)	
Normal gestational age, n (%)	1327(76.0)	1364(77.9)	2177(76.8)	
LGA, n (%)	242(13.9)	212(12.1)	334(11.8)	
VLGA, n (%)	104(6.0)	86(4.9)	164(5.8)	

Data are presented as mean ± SD or n (%)

*VSGA* Very small-for-gestational-age (< 3rd percentile), *SGA* Small-for-gestational-age (< 10th percentile), *LGA* Large-for-gestational-age (> 90th percentile), *VLGA* Very large-for-gestational-age (> 97th percentile), *LBW* Low birthweight (< 2500 g), *HBW* High birthweight (> 4000 g)

^a^One-way ANOVA

^b^Pearson chi-square test

**Table 3:** Adjusted odds ratios for low birthweight and small-for-gestational-age in singleton births by gestational age and endometrial thickness change

	EMT Decrease	EMT stable	EMT Increase
Gestational age ≥ 37 weeks			
SGA (< 10th percentile)			
AOR (95% CI)	1.141(0.783–1.662)	Reference	0.912(0.660–1.261)
Large for gestational age (> 90th percentile)			
AOR (95% CI)	0.822(0.676-1.000)	Reference	0.950(0.793–1.138)
LBW(< 2500 g)			
AOR (95% CI)	1.645(0.818–3.307)	Reference	1.310(0.723–2.375)
High birth weight (> 4000 g)			
AOR (95% CI)	0.784(0.600-1.025)	Reference	0.872(0.680–1.117)
Gestational age < 37 weeks			
Small for gestational age (< 10th percentile)			
AOR (95% CI)	9.992(0.587–17.227)	Reference	0.893(0.090–8.898)
Low birth weight(< 2500 g)			
AOR (95% CI)	0.895(0.195–4.104)	Reference	0.087(0.019–0.388)

Models were adjusted for maternal age and BMI, paternal age, infertility cause, parity, infertility duration, FET cycle rank, endometrial preparation type, number of embryos transferred, newborn sex, and previous SGA newborns. The stable EMT group served as the reference

*aOR *adjusted odds ratio*, CI *Confidence interval*, LBW *Low birthweight*, SGA *Small-for-gestational-age

**Table 4:** Birthweight and weight categories stratified by gestational age at delivery and endometrial thickness change

	EMT Decrease	EMT stable	EMT Increase	*P* value
Gestational age ≥ 37 weeks				
N	1617	1640	2651	
Newborn gender, n (%)				0.505^b^
Female	788(48.7)	817(49.8)	1272(48.0)	
Male	829(51.3)	823(50.2)	1379(52.0)	
Mean birthweight (g)	3423.65 ± 424.872	3400.65 ± 418.856	3410.05 ± 423.527	0.294^a^
Normal weight, n (%)	1439(89.0)	1485(90.5)	2390(90.2)	0.222^b^
LBW, n (%)	18(1.1)	24(1.5)	32(1.2)	
HBW, n (%)	145(9.0)	109(6.6)	200(7.5)	
VHBW, n (%)	15(0.9)	22(1.3)	29(1.1)	
VSGA, n (%)	23(1.4)	28(1.7)	42(1.6)	0.807^b^
SGA, n (%)	44(2.7)	49(3.0)	91(3.4)	0.402^b^
LGA, n (%)	230(14.2)	204(12.4)	323(12.2)	0.096^b^
VLGA, n (%)	103(6.4)	80(4.9)	147(5.5)	0.178^b^
Gestational age < 37 weeks				
N	129	110	184	
Newborn gender, n (%)				0.732^b^
Female	64(49.6)	49(44.5)	88(47.8)	
Male	65(50.4)	61(55.5)	96(52.2)	
Mean birthweight (g)	2498.57 ± 605.965	2615.59 ± 629.418	2396.39 ± 732.408	0.025^a^
Normal weight, n (%)	74(57.4)	65(59.1)	86(46.7)	0.067^b^
VLBW, n (%)	9(7.0)	4(3.6)	24(13.0)	
LBW, n (%)	46(35.7)	39(35.5)	73(39.7)	
HBW, n (%)	0(0.0)	1(0.9)	1(0.5)	
VHBW, n (%)	0(0.0)	1(0.9)	0(0.0)	
VSGA, n (%)	3(2.3)	5(4.5)	7(3.8)	0.632^c^
SGA, n (%)	6(4.7)	11(10)	27(14.7)	0.017^c^

Data are presented as mean ± SD or n (%)

*LBW* Low birthweight (< 2500 g), *VLBW* Very low birthweight (< 1500 g), *HBW* High birthweight (> 4000 g), *VHBW* Very high birthweight (> 4500 g), *VSGA* Very small-for-gestational-age (< 3rd percentile), *SGA* Small-for-gestational-age (< 10th percentile), *LGA* Large-for-gestational-age (> 90th percentile), *VLGA* Very large-for-gestational-age (> 97th percentile)

^a^One-way ANOVA

^b^Pearson’s chi-square test

^c^Fisher’s exact test

### Multivariate analysis

Multivariate linear regression analysis showed that after adjusting for confounders, EMT change was not independently associated with birth weight (Table 5). Neonatal sex (β = 130.1 g, *p* < 0.001) and gestational age at delivery (β = 172.6 g/week, *p* < 0.001) were independent determinants of birth weight.

**Table 5:** Multiple linear regression analysis of determinants of birthweight in live-born singletons

Model	Non-standardized coefficients		Standardized coefficients	t	*P* value
	B	Std. error	Beta		
Constant	-3335.343	146.076		-22.833	0.000
FET cycle rank, High order (vs. First)	6.485	10.922	0.006	0.594	0.553
Maternal age (y)	0.115	1.986	0.001	0.058	0.954
Paternal age (y)	0.179	1.484	0.002	0.121	0.904
Duration of infertility(y)	-3.046	2.054	-0.016	-1.483	0.138
Parity, High order(vs. First )	-8.629	10.794	-0.009	-0.799	0.424
Maternal BMI (kg/m^2^)	-0.655	1.767	-0.004	-0.370	0.711
FET endometrial preparation, Artificial cycle (vs. Ovulatory cycle)	-12.873	11.218	-0.012	1.148	0.251
EMT_*P*−1_ (mm)	9.462	4.925	0.041	1.921	0.055
EMT_ET day_ (mm)	-10.407	4.545	-0.051	-2.290	0.022
EMT change					
Remain stable(reference)					
Increased	-22.158	15.251	-0.022	-1.453	0.146
Decreased	1.561	15.245	0.001	0.102	0.918
No. of embryo transferred	5.084	18.880	0.003	0.269	0.788
Pregnancy-related complications, yes (vs. no)	-23.406	19.387	-0.013	-1.207	0.227
Infant gender boys (vs. girls)	130.122	10.362	0.130	12.558	0.000
Gestational age (weeks)	172.641	3.275	0.560	52.707	0.000

*EMT*_P−1_ denotes the endometrial thickness (mm) on the day preceding progesterone initiation, and *EMT*_ET day_ represents the endometrial thickness (mm) on the day of embryo transfer

### Subgroup analysis by endometrial preparation protocol

Given documented hormonal differences between natural cycles (NC) and estrogen-progesterone (EP) cycles—particularly the presence or absence of corpus luteum-derived vasoactive hormones and distinct estrogen exposure patterns—we formally assessed effect modification by preparation protocol.

Interaction terms (EMT change × cycle type) were added to multivariable models for all three primary outcomes. These interactions were not statistically significant for SGA (Wald χ² = 0.744, df = 2, *P* = 0.689), continuous birthweight (EMT decrease × EP: β = -9.71 g, 95% CI -61.02 to 41.60, *P* = 0.711; EMT increase × EP: β = +5.10 g, 95% CI -41.83 to 52.03, *P* = 0.831), or LBW (Wald χ² = 0.854, df = 2, *P* = 0.652).

Stratified analyses showed consistent effect directions across protocols. For birthweight, an EMT increase was associated with lower weight in both NC (β = -35.86 g, *P* = 0.009) and EP cycles (β = -30.76 g, *P* = 0.024). For SGA and LBW, point estimates were directionally concordant, though individually non-significant in stratified analyses (Supplement Table 2).

## Discussion

In this retrospective cohort analysis of 6,331 live-born singletons after FET, we present preliminary evidence that progesterone-induced alterations in EMT do not significantly affect mean neonatal birthweight. Furthermore, these EMT changes were not linked to altered risks of PTB, LBW, SGA, or term pregnancy complications. After adjusting for multiple confounders, infant sex (*P* < 0.001) and gestational age (*P* < 0.001) were independent determinants of birthweight.

This study provides progesterone-induced EMT changes do not affect singleton infant outcomes during frozen-thawed embryo transfer (FET) cycles. While prior research from our group showed no effect on clinical pregnancy or live birth rates, this analysis extends the findings to perinatal outcomes in over 6,000 singleton newborns. Unlike earlier studies that measured thickness at a single proliferative-phase point, we evaluated dynamic responses to progesterone-a parameter reflecting secretory transformation rather than estrogen-driven proliferation. The results for term infants, along with an exploratory observation of lower Z-scores in the group with increased thickness, suggest endometrial compaction may be a benign physiological variation without clinical impact on fetal growth, though prospective validation is required.

In recent decades, significant research has examined how EMT affects IVF success rates. Many studies indicate that a thin endometrial lining adversely affects pregnancy outcomes [11, 22, 38, 39]. However, evidence on the potential link between EMT and neonatal birthweight remains limited. An initial study by Chung et al. investigated factors affecting adverse perinatal outcomes following IVF, reporting a twofold increased risk in individuals with EMT ≤ 10 mm compared to those with EMT > 12 mm (OR: 2.04; 95% CI: 1.09–3.83) [40]. Additionally, greater endometrial thickness showed a significant protective effect on infant outcomes (OR: 0.89; 95% CI: 0.80–0.99). Each millimeter decrease in thickness was associated with a 12% relative increase in the risk of adverse perinatal outcomes [40]. However, no statistically significant risk difference was found among singletons (aOR: 1.66, 95% CI: 0.73–3.81) [40]. Our findings differ from those of Chung et al., who reported a 12% relative rise in adverse perinatal outcomes per 1 mm reduction in endometrial thickness. Potential explanations include: (1) Chung et al. measured EMT during ovarian stimulation in fresh cycles, where supraphysiological estradiol may confound the association; (2) their outcome was a composite of obstetric complications, not specific fetal growth measures; and (3) our FET cohort eliminates ovarian stimulation confounders, isolating the endometrial response to progesterone. Another study reported that endometrial thickness on the day of HCG trigger positively correlated with neonatal birthweight, especially in pregnancies with obstetric complications [41]. This association was not significant in uncomplicated pregnancies [41]. Oron et al. found that an endometrial thickness < 7.5 mm significantly increased the risk of adverse obstetric outcomes (adjusted OR: 1.53; 95% CI: 1.03–2.42; *P* = 0.04), though no significant effect on singleton birth weight was observed [16]. Similarly, Du et al. found that an EMT ≤ 7.5 mm was linked to higher low birthweight risk in 2,847 fresh-cycle singletons [42]. In contrast, our analysis of FET cycles with controlled endometrial preparation shows that dynamic EMT changes-not absolute thickness-do not predict neonatal outcomes. This underscores a key nuance: proliferative-phase EMT may be predictive, while progesterone-induced changes are not. A recent large-scale retrospective study of 5,220 singleton newborns found that an EMT below 8 mm was associated with an adjusted odds ratio (aOR) of 1.57 (95% CI: 1.09–2.26) for low birth weight in FET cycles [43]. This result aligns with prior research from our institution [21, 31].

In most previous studies, EMT was evaluated during the proliferative phase before progesterone administration. However, endometrial development differs physiologically between the follicular and luteal phases. During the follicular phase, estrogen promotes EMT and stimulates linear growth of endometrial glands and blood vessels [23]. After ovulation, progesterone halts endometrial proliferation within 2–3 days [23]. Little research has examined how changes in EMT following progesterone administration affect pregnancy outcomes and neonatal health.

Although some studies have examined changes in EMT during IVF stimulation, their results are inconsistent [24, 25, 27, 44–46]. Zhao et al. found that neither the change in endometrial thickness (EMT) from stimulation day 3 to the hCG administration day, nor its combination with EMT type, significantly predicted pregnancy or clinical outcomes [27]. However, they did not examine whether EMT changes after progesterone administration correlate with pregnancy and neonatal outcomes. Haas et al. investigated EMT changes between the late estrogen phase and embryo transfer day in 274 FET cycles, finding a strong inverse correlation between EMT changes and pregnancy outcomes [44]. However, the study had limitations: EMT was measured via transabdominal ultrasound on transfer day versus vaginal ultrasound at the estrogen phase end; only women on hormonal preparation protocols were included; and it focused solely on ongoing pregnancy rates without exploring links between post-progesterone EMT changes and neonatal outcomes. Another study found that progesterone administration increased endometrial thickness, which correlated with better pregnancy outcomes [45]. However, the link between such thickness changes and neonatal outcomes remains unexplored. Our previous research showed that changes in endometrial thickness after progesterone use did not significantly affect clinical pregnancy or live birth rates in FET cycles [32], yet we also did not investigate their association with neonatal outcomes. This study extends our prior research by: (1) directly linking progesterone-induced endometrial changes to neonatal outcomes, beyond just pregnancy establishment; (2) using FET cycles to isolate endometrial factors from ovarian stimulation effects; and (3) demonstrating that statistically detectable EMT changes lack clinically meaningful prognostic value for term infant growth.

This study addressed limitations of prior research by examining the precise effect of progesterone administration on EMT change and its relationship with neonatal outcomes in FET cycles. Based on over 6000 singleton newborns conceived via FET, our findings show that any progesterone-induced EMT change does not significantly affect term newborn outcomes. The absence of association between progesterone-induced EMT changes and birthweight has key mechanistic implications. Normal secretory transformation involves endometrial compaction from stromal edema resolution and glandular coiling under progesterone. Our findings indicate that the extent of this compaction-whether minimal (stable EMT) or more pronounced (reduced EMT)-does not alter the intrauterine environment enough to impact fetal growth. This suggests endometrial thickness may be a marker of receptivity, not a determinant of placental function or nutrient transfer capacity. These findings provide observational reassurance for clinical practice: patients with EMT changes after progesterone administration can be counseled that current evidence does not support an independent association with adverse neonatal outcomes in term singletons. However, individualized assessment remains warranted, particularly for patients with extreme EMT changes or additional risk factors.

The three groups showed significant differences in baseline and cycle characteristics, such as parity, fertility duration, FET endometrial preparation, and number of embryos transferred. However, linear regression analysis indicated that these factors did not affect neonatal birthweight. Further analyses accounting for pregnancy outcomes also yielded consistent results. Some adjusted odds ratios had wide confidence intervals (e.g., preterm SGA: aOR 0.893, 95% CI 0.090–8.898), reflecting limited precision due to low event numbers; these estimates should be considered inconclusive rather than evidence of no effect. In contrast, the null findings for term infants are more robust, supported by larger sample sizes and narrower confidence intervals. The present study used multiple linear regression to confirm that birthweight is predicted by newborn gender and gestational age, consistent with prior literature [21, 47, 48]. Male neonates are predicted to weigh 130 g more than females, and each additional week of gestation is associated with a 172 g increase in birthweight. To account for potential biases from gender and gestational age, Z-scores were calculated and compared across the three groups, consistently showing lower scores in EMT increase group. Despite similar mean birthweights across EMT change groups, Z-scores were significantly lower in the EMT increase group (0.34 ± 1.07) than in the decrease group (0.43 ± 1.04; *p* = 0.007). This difference is clinically relevant, as Z-scores adjust for gestational age and sex, unlike raw birthweight. The lower Z-scores in the increase group indicate a subtle trend toward relatively smaller fetal size for gestational age, despite a non-significant absolute difference (~ 90 g). However, caution is warranted: (1) the effect size is small (~ 0.09 SD); (2) the clinical importance of such a minor difference is unclear; and (3) multiple comparisons raise the risk of Type I error. Still, this finding suggests that persistent endometrial expansion (lack of compaction) may indicate subtle progesterone resistance, meriting exploration in prospective studies with standardized luteal phase hormone assessments.

Several large studies and meta-analyses indicate that NC and EP frozen embryo transfer yield similar pregnancy rates in ovulatory women [49]. Some evidence suggests NC cycles may lower the risk of hypertensive disorders and preterm birth [50]. However, the underlying endometrial changes likely differ biologically: in NC cycles, they result from endogenous luteal progesterone, while in EP cycles, they reflect a response to exogenous progesterone in an estrogen-primed endometrium [51]. To address potential confounding by cycle type, we included endometrial preparation protocol as a covariate in all multivariable models. Sensitivity analyses stratified by NC versus EP cycles revealed no significant interaction with EMT change, supporting the combination of these protocols. Stratified analyses showed consistent effect directions, validating the inclusion of both NC and EP cycles in the primary analysis. Our finding of comparable EMT changes across protocols contrasts with studies reporting divergent birthweight outcomes by preparation protocol [35]. The absence of significant interaction suggests progesterone-induced EMT changes may represent a universal endometrial response rather than a protocol-specific effect. However, wider confidence intervals in stratified analyses, particularly for EP cycles (about one-third of the cohort), indicate reduced precision. Although the absence of corpus luteum-derived vasoactive hormones (e.g., relaxin) in EP cycles has been hypothesized to increase birthweight and LGA risk [34], our data do not suggest this modifies the relationship between EMT change and neonatal outcomes. The lack of meaningful effect modification implies that progesterone-induced EMT changes influence neonatal outcomes through a common biological mechanism-likely related to endometrial secretory transformation and subsequent placental development-regardless of endogenous or exogenous hormonal preparation.

This study has limitations. While the database was carefully reviewed using strict criteria, the retrospective design inherently restricts the interpretation of the results. To minimize potential confounding by culture duration on FET outcomes, the analysis was limited to neonatal outcomes following day 3 embryo transfers [52]. This restriction introduces a potential selection bias, as the cohort included only singleton live births after Day 3 FET cycles, excluding biochemical pregnancies, miscarriages, stillbirths, and multiple gestations. Consequently, if EMT changes mainly affect early pregnancy loss rather than fetal growth, such effects would not be detected in an analysis conditional on live birth. Additionally, limiting the study to women aged ≤ 40 years and with a body mass index (BMI) < 30 kg/m² may reduce the generalizability of the findings to higher-risk populations. Regarding measurement, EMT was assessed by a single experienced operator using standardized equipment, though the retrospective design prevented operator blinding to outcomes. The absence of formal inter-observer reliability testing in this dataset is a limitation, although prior validation at this center reported an intraclass correlation coefficient (ICC) > 0.90 for EMT measurements [53]. Additionally, EMT was measured at only two time points; continuous monitoring could reveal more nuanced patterns. A key strength is the single-center design, which minimized inter-center variability in endometrial preparation, ultrasound techniques, and neonatal assessments. Throughout the study (2010–2019), the hormonal regimen and FET protocols were applied consistently. Nevertheless, subtle temporal changes in practice-such as shifts in embryo transfer rates-did occur over the decade. These were addressed by adjusting for FET cycle rank and treatment year in multivariable models.

### Conclusions and future prospects

This large-scale single-center study offers valuable insights into how progesterone-induced endometrial thickness changes affect pregnancy outcomes in frozen embryo transfer cycles. The findings indicate that these changes are unlikely to significantly influence neonatal birthweight. Although observationally reassuring for clinical practice, the retrospective design and lack of randomized intervention limit causal conclusions. Patients with such changes can be informed that current evidence does not show an independent link to adverse neonatal outcomes, though individualized assessment is still advised.

Nevertheless, longer prospective cohort studies are needed to confirm these findings. Future research should use a composite outcome framework that combines implantation-phase and perinatal endpoints. More specifically, prospective studies should: (1) determine whether changes in EMT predict not only pregnancy establishment (clinical pregnancy, ongoing pregnancy, and live birth) but also later perinatal outcomes like birthweight, SGA, and LBW within the same cohort; (2) examine whether EMT changes are linked to first-trimester biomarkers of placental development (e.g., PAPP-A, free β-hCG), which may mediate effects on fetal growth; and (3) explore whether luteal-phase progesterone levels influence the relationship between EMT changes and neonatal outcomes, as progesterone resistance—manifested as insufficient EMT compaction—may represent a distinct endometrial phenotype affecting both implantation and placental function.

## Supplementary Information


Supplementary Material 1.


## Data Availability

The data that support the findings of this study are available from the corresponding author upon reasonable request.

## Abbreviations

EMT: Endometrial Thickness
FET: Frozen-thawed Embryo Transfer
IVF: In vitro Fertilization
ART: Assisted Reproductive Technology
SGA: Small Gestational Age
VSGA: Very small for Gestational Age
LGA: Large for Gestational Age
VLGA: Very Large for Gestational Age
LBW: Low Birthweight
VLBW: Very Low Birthweight
HBW: High Birthweight
VHBW: Very High Birthweight
PTB: Preterm Birth
