# Supplementary material for: Progesterone-induced endometrial thickness changes do not affect singleton neonatal outcomes in frozen embryo transfer: a retrospective analysis of 6,331 newborns
Source: BMC Pregnancy Childbirth. 2026 Jun 20;26:971. doi: 10.1186/s12884-026-09391-9 (PMC13528165; doi:10.1186/s12884-026-09391-9)
Supplement: Supplementary file 1 [file 12884_2026_9391_MOESM1_ESM.docx]

**Supplement Table1 Collinearity diagnostics**

| **Variable** | **Tolerance** | Variance inflation factors (VIF) | **Diagnosis** |
| --- | --- | --- | --- |
| ΔEMT group | 0.989 | 1.011 | No multicollinearity |
| FET rank | 0.99 | 1.01 | No multicollinearity |
| Maternal age | 0.476 | 2.1 | No multicollinearity |
| Paternal age | 0.499 | 2.004 | No multicollinearity |
| Infertility duration | 0.876 | 1.142 | No multicollinearity |
| Maternal BMI | 0.989 | 1.012 | No multicollinearity |
| GA weeks | 0.992 | 1.008 | No multicollinearity |
| Parity | 0.939 | 1.065 | No multicollinearity |
| Newborn sex | 0.997 | 1.003 | No multicollinearity |
| Previous SGA | 0.994 | 1.006 | No multicollinearity |
| Embryo number | 0.998 | 1.002 | No multicollinearity |

Note: A VIF (Variance Inflation Factor) less than 5.0 indicates no multicollinearity; a tolerance greater than 0.1 indicates strong independence among variables.

**Supplement Table 2 The relationship between EMT changes, categorized by cycle type, and neonatal outcomes**

| Outcome | EMT change | NC cycle | | EP cycle | | Interaction |
| --- | --- | --- | --- | --- | --- | --- |
|  |  | Effect (95% CI) | P | Effect (95% CI) | P | P value |
| Birthweight (g) | Increase vs Stable | -35.86 (-62.72 ~ -9.01) | 0.009 | -30.76 (-58.52 ~ -3.00) | 0.024 | 0.831 |
|  | Decrease vs Stable | +14.34 (-16.08 ~ 44.77) | 0.355 | +4.64 (-25.79 ~ 35.07) | 0.762 | 0.711 |
| SGA | Increase vs Stable | 0.828 (0.409 ~ 1.675) | 0.599 | 0.919 (0.432 ~ 1.954) | 0.825 | 0.813 |
|  | Decrease vs Stable | 0.864 (0.433 ~ 1.721) | 0.677 | 0.647 (0.273 ~ 1.534) | 0.324 | 0.51 |
| LBW | Increase vs Stable | 1.478 (0.636 ~ 3.436) | 0.364 | 0.945 (0.377 ~ 2.367) | 0.902 | 0.404 |
|  | Decrease vs Stable | 1.193 (0.508 ~ 2.801) | 0.685 | 0.762 (0.268 ~ 2.169) | 0.612 | 0.99 |

Note: Birth weight is presented as the linear regression coefficient (β), SGA and LBW are reported as adjusted odds ratios (aOR). P values for interaction are derived from tests of the interaction terms within the model.

**Supplement Table III Birth weights and birth weights categories of live born singletons**

|  | **EMT Decrease (n=1746)** | **EMT stable (n=1750)** | **EMT Increase (n=2835)** | **P value** |
| --- | --- | --- | --- | --- |
| Normal weight, 2500–4000 g, n (%) | 1513(86.7) | 1550(88.6) | 2476(87.3) | 0.070^b^ |
| LBW (< 2500 g), n (%) | 64(3.7) | 63(3.6) | 105(3.7) |  |
| HBW (> 4000 g), n (%) | 145(8.3) | 110(6.3) | 201(7.1) |  |
| VHBW (> 4500 g), n (%) | 15(0.9) | 23(1.3) | 29(1.0) |  |
| VLBW (< 1500 g), n (%) | 9(0.5) | 4(0.2) | 24(0.8) |  |

LBW, low birth weight; VLBW, very low birth weight; HBW, high birth weight; VHBW, very high birth weight.

^b^Pearson chi-square test. Values are number (percentage).

Values are number (percentage).

**Supplement Table IV Crude ORs for birth weights and birthweights categories in singleton births by gestational age.**

|  | **EMT Decrease** | **EMT stable** | **EMT Increase** |
| --- | --- | --- | --- |
| **Gestational age≥37 weeks** | | | |
| **Small for gestational age (<10th percentile)** |  |  |  |
| Crude OR (95% CI) | 1.140(0.815-1.593) | Reference | 0.933(0.700-1.244) |
| **Large for gestational age (>90th percentile)** |  |  |  |
| Crude OR (95% CI) | 0.808(0.677-0.963) | Reference | 0.972(0.826-1.143) |
| **Low birth weight(<2500g)** |  |  |  |
| Crude OR (95% CI) | 1.319(0.713-2.440) | Reference | 1.216(0.713-2.071) |
| **High birth weight (>4000g)** |  |  |  |
| Crude OR (95% CI) | 0.791(0.621-1.007) | Reference | 0.918(0.734-1.149) |
| **Gestational age < 37 weeks** | | | |
| **Small for gestational age (<10th percentile)** |  |  |  |
| Crude OR (95% CI) | 2.278(0.814-6.376) | Reference | 0.646(0.307-1.361) |
| **Low birth weight(<2500g)** |  |  |  |
| Crude OR (95% CI) | 0.864(0.514-1.450) | Reference | 0.576(0.356-0.930) |

CI: confidence interval, OR: odds ratio,

Analyses were adjusted for maternal age and BMI, paternal age, infertility cause, parity, infertility duration, FET cycle rank, the type of endometrial preparation, number of embryos transferred, newborn sex, previous SGA newborns.
